# Liver Maximum Capacity (LiMAx) Test Is a Promising Tool to Predict Fibrosis and Cirrhosis in Chronic Liver Disease: A Systematic Review

**DOI:** 10.7759/cureus.94173

**Published:** 2025-10-09

**Authors:** Anushri Joshi, Eashan Patel

**Affiliations:** 1 Ear, Nose and Throat, West Suffolk National Health Service (NHS) Foundation Trust, Bury St Edmunds, GBR; 2 Trauma and Orthopaedics, West Hertfordshire Teaching Hospitals National Health Service (NHS) Trust, West Hertfordshire, GBR

**Keywords:** chronic liver disease, cirrhosis, cps, fibrosis, limax, liver maximum functional capacity, meld, mortality, steatosis

## Abstract

Patients affected with chronic liver disease (CLD) are increasing and our ability to manage them is hindered by the lack of a simple and accurate test for measuring the liver function. LiMAx is a novel, non-invasive tool that measures the metabolic capacity of hepatocytes by measuring the ratio of ^13^CO_2_ to ^12^CO_2 _exhaled following the administration of isotope-labelled methacetin. This systematic review assessed the ability of LiMAx to diagnose and stage steatosis and fibrosis and predict 90-day mortality in CLD. Literature search was carried out using Embase and Medline accessed via Ovid, Web of Science, and Cochrane Library. The inclusion criteria were case-control or cohort studies in all languages. Statistical analysis for the diagnostic accuracy of LiMAx and the diagnostic accuracy of other available methods for clinically relevant milestones in CLD was carried out by pooling the area under the receiver operating characteristic curve (AUROC) and correlation coefficients. A total of seven studies met the inclusion criteria and data were extracted into Microsoft Excel tables with a total number of 1623 participants. LiMAx performed significantly better than transesophageal echocardiography (TE), Fibrosis-4 (FIB-4) test, aspartate transaminase to alanine transaminase ratio (AAR), spleen size and aspartate aminotransferase-to-platelet ratio index (APRI) at predicting 90-day mortality (AUROC=0.82). LiMAx is superior in detecting cirrhosis (AUROC=0.92) and at identifying fibrosis for patients with non-MASLD (metabolic dysfunction-associated steatotic liver disease) aetiology. Furthermore, LiMAx had the strongest (negative) correlation with liver histopathology (r=-0.75). It was found that the LiMAx test is a promising point-of-care approach for the detection of cirrhosis and fibrosis. However, large-scale studies across all liver aetiologies and stages are necessary to confirm the utility of LiMAx in clinical practice.

## Introduction and background

Despite falling mortality rates for other chronic diseases, the mortality rate of chronic liver disease (CLD) in the UK has dramatically increased in recent decades (400% since the 1970s) [1]. CLD develops on the background of genetic and environmental factors, with the majority arising from lifestyle and metabolic syndrome driving hepatic steatosis, inflammation and fibrosis. It is defined as the deterioration in liver function for six months or longer [2]. One in three people is estimated to have metabolic-associated steatotic liver disease (MASLD), which is the presence of hepatic steatosis in individuals with at least one metabolic risk factor and minimal or no alcohol consumption. A further 5% of the population have progressed to metabolic dysfunction-associated steatohepatitis (MASH), making them susceptible to fibrosis. Early identification is crucial; as histological changes are reversible at this stage with lifestyle changes. Weight loss of 10% can improve liver function in MASLD and MASH [3]. In advanced stages, where CLD leads to systemic complications such as portal hypertension, impaired liver function correlates with increased mortality. Therefore, precise assessment of liver function is essential to guide effective treatment strategies.

A significant obstacle in the clinical management of CLD is the absence of a straightforward and accurate modality for quantifying hepatic function. Although liver biopsy remains the diagnostic gold standard, its utility is limited to histopathological evaluation rather than functional assessment. Furthermore, it is invasive and subject to sampling variability, which may compromise diagnostic accuracy [4]. Non-invasive radiological techniques aimed at estimating hepatic reserve are increasingly employed; however, their reliability is diminished in certain patient populations, such as individuals with obesity, and they offer a limited insight into the qualitative and functional status of hepatic tissue [5,6]. Composite scoring systems, including the Child-Pugh Score (CPS) and the Model for End-Stage Liver Disease (MELD), incorporate clinical parameters and biochemical markers to stratify disease severity. Nevertheless, these tools are constrained by the inclusion of subjective components (such as the clinical assessment of ascites and hepatic encephalopathy), which may introduce variability and reduce prognostic precision [7,8].

This underscores a critical need for a more precise and responsive modality capable of capturing the dynamic physiological changes inherent to the progression of chronic liver disease. The existing diagnostic approaches are demonstrably suboptimal and, as such, are insufficient when used in isolation for comprehensive assessment or therapeutic decision-making. The LiMAx test, short for liver maximum capacity, is a specialised breath test used to measure how well the liver is functioning in real time. LiMAx is a novel point-of-care diagnostic breath test that uses a liver-specific enzymatic pathway to quantitatively estimate hepatic function by measuring the ratio of ^13^CO_2_ to ^12^CO_2 _in breath [9]. The patient is intravenously administered ^13^C-labelled methacetin, which is metabolised by the cytochrome P450 enzyme (CYP1A2) into paracetamol and CO_2_. The labelling is done by isotope substitution to allow for tracking of the methacetin and its metabolites. The exhaled CO_2_ is measured using breath analysis by the LiMAx FLIP medical device (Humedics GmbH, Berlin, Germany). The ratio of labelled (^13^C) to unlabelled (^12^C) CO_2_ correlates with the level of CYP1A2 activity and acts as a marker of liver function.

The use of this test has been examined in patients undergoing liver resection or transplant for better selection of patients [10-14]. LiMAx has been introduced into clinical practice at some of the European centres for evaluating non-surgical patients with CLD [15], but its role is not fully established. The National Institute for Health and Care Excellence (NICE) has previously evaluated the use of LiMAx for guiding decisions in liver surgery and transplantation, particularly in predicting post-operative outcomes and assessing residual liver function. However, its potential role in diagnosing and managing cirrhosis within medical settings remains unexplored [15]. Therefore, we conducted a systematic review to evaluate the clinical utility of the LiMAx test in CLD, with a specific focus on its role in predicting hepatic fibrosis or cirrhosis, as well as its prognostic value in estimating 90-day mortality.

## Review

Materials and methods

Study Selection

A systematic literature search was performed in Embase, Medline, Web of Science, and Cochrane. Medline and Embase were accessed via Ovid. The date range of publications searched was between 1946 and present.

The search terms were as follows (“13C-methacetin breath test” OR “LiMAx” OR “Liver Maximum Capacity”) AND (“Hepatic function” OR “Hepatic reserve” OR “Liver reserve” OR “Liver Function” OR Hepatocellular dysfunction” OR “Hepatocellular injury”) AND (“Cirrho*” OR “Fibro*” OR “CLD” OR “Chronic liver disease”). The last search was performed in February 2022 and was applied separately to individual databases and the results were compiled for further screening. To complement the search, the references included within the retrieved literature were also reviewed for any further articles that may have been appropriate for the aims of the review. A date range and language criteria were not applied as it would place limitations on the retrieval of already scarce data. The inclusion and exclusion criteria are shown in Table 1. Composite scores of LiMAX were included such as CreLiMax, a variation of the LiMAx score that combines creatinine levels with the LiMax test in a statistical model.

**Table 1:** Summary of the inclusion and exclusion criteria for the CLD review. LiMAx: Liver Maximum Capacity

	Inclusion Criteria	Exclusion Criteria
Study Design	Randomised Controlled Trials Clinical Trials Observational studies (Sample n>5)	Literature reviews, Poster presentations, Book chapters, Conference papers, Conference abstracts (Sample n<5)
Participant Characteristics	Participants have cirrhosis, fibrosis and/ or chronic liver disease Human Main morbidity is liver disease	Participants do not have cirrhosis, fibrosis and/or chronic liver disease; other concurrent comorbidities, acute liver failure, acute on chronic liver failure
Test Administered	LiMAx values present	Fails to mention LiMAx
Comparator	At least one non-imaging comparator present	No comparator or non-imaging comparator

The selected papers underwent a complete independent review by two investigators (A.J. and E.P.) and disagreements were discussed.

Quality Assessment and Data Extraction

All studies were assessed using the Quality Assessment of Diagnostic Accuracy Studies (QUADAS-2) [16]. The following variables were extracted from each study, if available: authors, year of publication, country, and study design. The baseline characteristics for each of the populations were collected, including the number of participants, average age, male-to-female ratio, and proportion of CLD aetiologies. Then for the chosen aim (ability to predict 90-day mortality; cirrhosis and fibrosis), the area under the receiver-operating characteristic (AUROC) values were recorded when provided by the studies. In addition to this, correlation values of liver tests to LiMAx and stages of CLD were recorded where available.

Statistical Analysis

Review Manager 5.4 (RevMan), designed by Cochrane (The Cochrane Collaboration, London, UK) for the purposes of meta-analyses was used [17]. Statistical analyses for the CLD review were focused on pooling the AUROCs and pooling correlation coefficients. AUROC pooling required AUROCs and their corresponding standard error (SE) values, which were calculated from the 95% confidence intervals (CI) if given or by using a formula described by Hanley et al. [18,19]. In the event of missing data, the number of events/non-events had to be estimated before the SE could be calculated. For these studies, the total number of included patients was provided, so it was assumed that the missing data was distributed at random. This meant that the patients in the events/non-events group were equally as likely to have missing data and there was no selection bias. Therefore, the number for the events/non-events groups was adjusted to give the total N, with the same relative proportion. RevMan would then calculate the pooled effect alongside a measure of heterogeneity between studies when more than one study reported AUROC values for a particular test, the I^2^, derived from the Chi-squared statistic, which measured the extent to which the ‘true effect’ size was compromised [20]. Microsoft Excel (Microsoft, Redmond, WA) was then used to generate forest plots of the pooled AUROCs [21].

The analysis of correlation coefficients (Spearman’s rho) was performed using the “meta” package in R (R Foundation for Statistical Computing, Vienna, Austria), which applied Fisher’s Z-transformation to the correlation coefficients and then pooled these using a random-effects inverse-variance model [20].

Results

**Figure 1:**
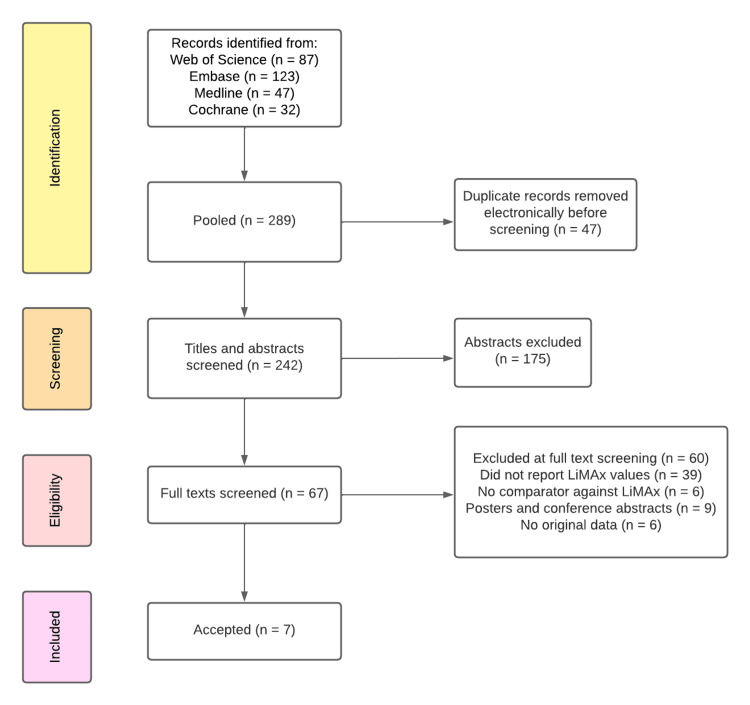
PRISMA diagram for CLD review to depict search strategy and reasons for exclusion at various steps. A total of seven studies included. PRISMA: Preferred Reporting Items for Systematic Reviews and Meta-Analyses.

A total of seven studies with 1683 patients were included in the review (Figure 1). Patient demographics from the papers are provided in Table 2 [22-28]. The age range included in these papers within two standard deviations (SD) or the interquartile range (IQR) is representative of a population affected by CLD in clinical practice, and pooling revealed a mean of 49.52 years (95% CI: 42.52, 56.52).

**Table 2:** Summary of patient demographics for CLD review. Data is presented as a mean±SD or as a median (IQR) NR: not reported; SD: standard deviation; IQR: interquartile range

Study	N	Age (Years)	BMI (kg/m^2^)	Sex M, n (%)
Jara et al. (2019) [22]	268	55 (49-60)	26.1 (23.0-29.4)	165 (61.6)
Dziodio et al. (2020) [23]	113	58 (51-63)	28.0 (23.9-31.1)	61 (54)
Buechter et al. A (2019) [24]	464	58.5±14.2	NR	279 (60.1)
Buechter et al. B (2019) [25]	102	48.6±16.7	NR	57 (55.9)
Schmitz et al. (2020) [26]	141	44 ±9	53 ±7	38 (27)
Malinowski et al. (2014) [27]	433	NR	NR	248 (57.3)
Group 1 (Child Pugh A)	86	28 (24-32)	23.0 (20.9-25.1)	48 (55.8)
Group 2 (Child Pugh B)	269	56 (49-61)	26.8 (23.6-29.7)	148 (68.4)
Group 3 (Child Pugh C)	78	56 (51-61)	25.9 (23.1-29.7)	52 (66.7)
Alizai et al. (2019) [28]	102	43.2±10.5	53.7±9.3	32 (31.3)

Consideration was given when interpreting the results of papers that solely looked at nonalcoholic fatty liver disease (NAFLD)-related aetiology. The assumption of normality was made for all studies as most of the data was summarised using mean values and SDs.

Ability to Predict 90-Day Mortality

**Figure 2:**
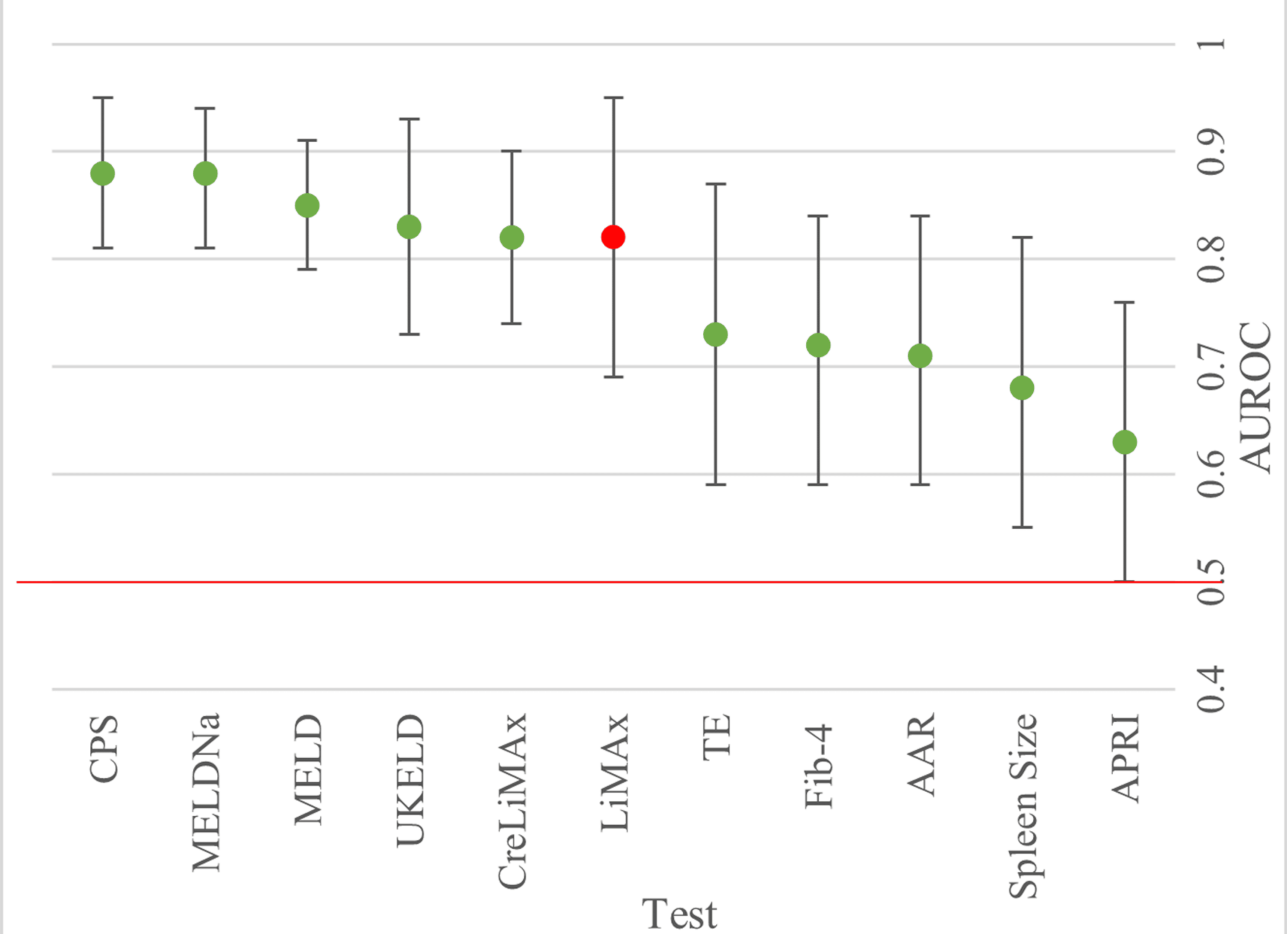
Forest plot displaying pooled AUROCs for the prediction of 90- day mortality. Cut-off line at 0.5 to show point at which there is no discrimination capacity. Table shows AUROC (area under the receiver operating curve) values plotted for each test and corresponding confidence interval (CI). * AUROC value from only one study available.

The 90-day mortality was reported in three studies [22-24]. After pooling the AUROCs for each test from the individual studies, a forest plot was made to compare the differences in accuracy (Figure 2).

LiMAx had a pooled AUROC value of 0.82 (95% CI: 0.69, 0.95) and performed significantly better than transient electrography (TE), Fibrosis-4 (FIB-4) index for liver fibrosis, aspartate transaminase to alanine transaminase ratio (AAR), spleen size and aspartate transaminase to platelet ratio (APRI) (the worst indicator) at predicting 90-day mortality. TE was the closest competitor to LiMAx and the best performer out of the tests using imaging. LiMAx compared well with the United Kingdom Model for End-Stage Liver Disease (UKELD) and CreLiMAx, whereas CPS and MELD performed better in predicting the 90-day mortality.

The pooling of MELD, MELDNa and CreLiMAx individually gave an I^2^ of 0, implying that there were no important differences that could distort the end result. I^2^ for the studies that were pooled for LiMAx and CPS values indicated that there may have been substantial heterogeneity present (I^2^=64% and 35%, respectively).

Ability to Detect Cirrhosis

**Figure 3:**
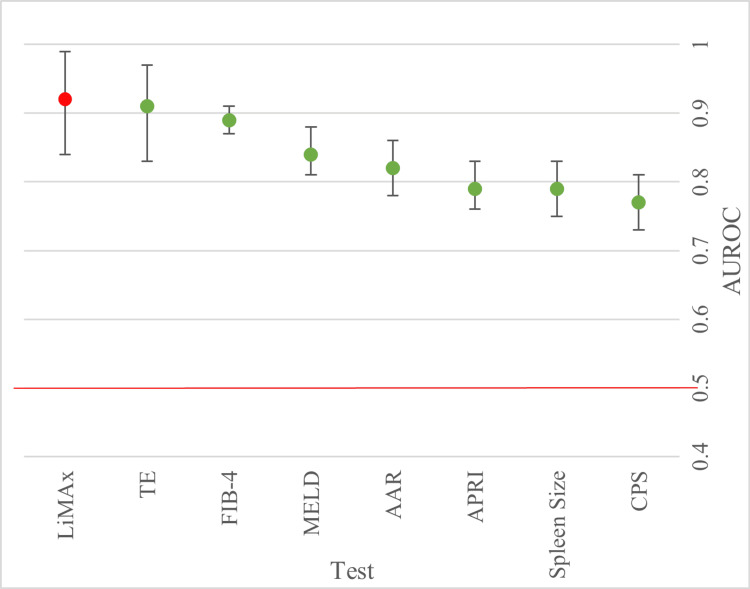
Forest plot displaying pooled AUROCs for the prediction for the ability to detect cirrhosis. Cut-off line at 0.5 to show point at which there is no discrimination capacity. AUROC: Area under the receiver operating curve.

This data shown in Figure 3 indicated that LiMAx is the superior measure of a cirrhotic liver, with an AUROC of 0.92 (95% CI: 0.84, 0.99). TE yielded an AUROC of 0.91 followed by the MELD score, which was able to detect cirrhosis with an AUROC of 0.84 (95% CI:0.81, 0.88), outperforming CPS, spleen size, APRI and AAR. The AUROC was lowest for CPS. LiMAx had the largest 95% confidence interval (0.85-0.99) and appreciably lower values were seen for the other scores as a general pattern of increasing spread in the AUROC data was seen for the tests that offered the most accuracy. However, there was a particular outlier with FIB-4 having the smallest CI (0.87-0.91) and a level of accuracy approaching LiMAx and TE.

Ability to Detect Fibrosis

**Figure 4:**
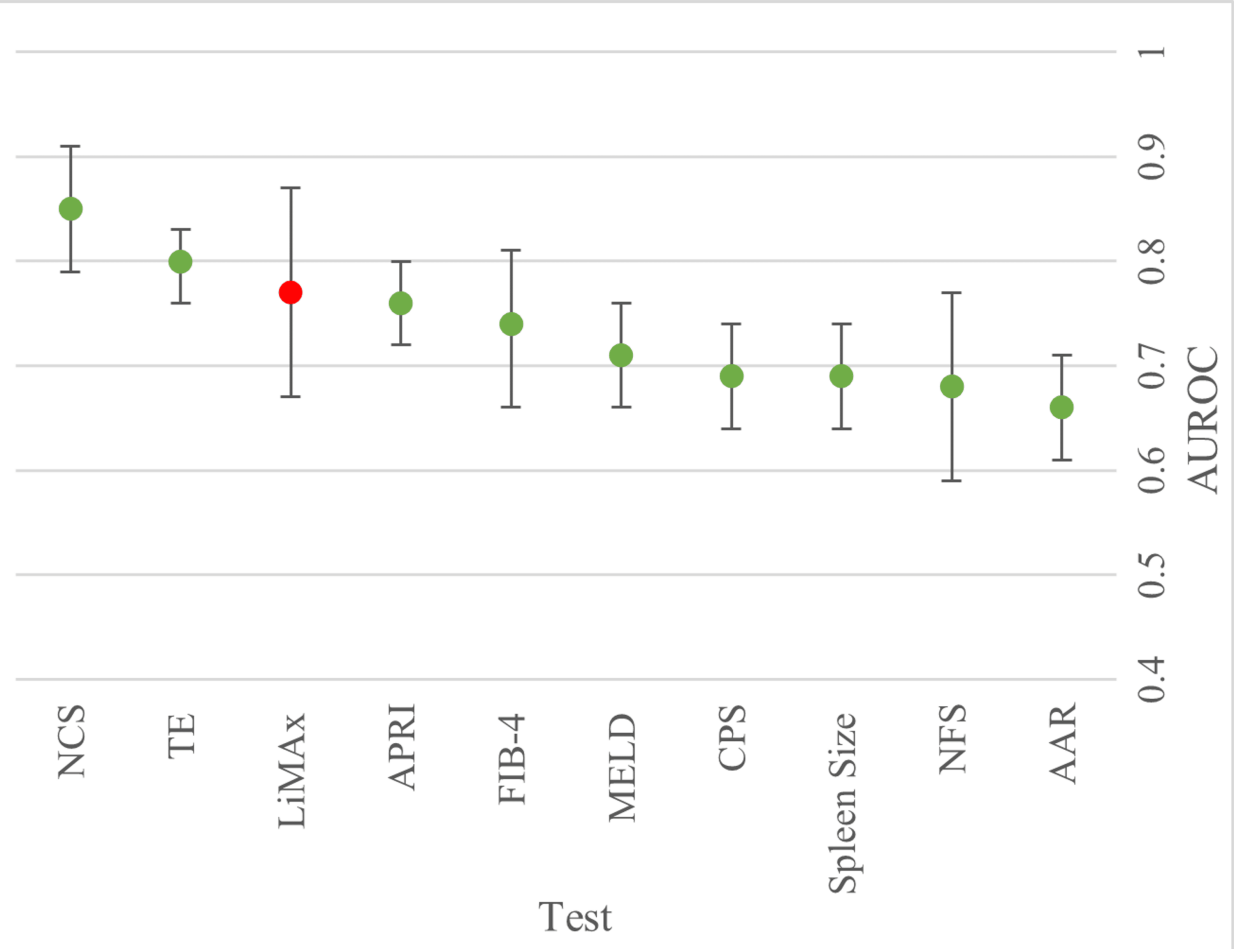
Forest plot displaying pooled AUROCs for the prediction for the ability to detect fibrosis. Cut-off line at 0.5 to show point at which there is no discrimination capacity. AUROC: Area under the receiver operating curve.

Three papers reported the ability to detect fibrosis [24-26]. Two of these papers, Schmitz et al. (2020) and Buechter at al. B (2019), explicitly stated that they were assessing the accuracy of the included tests for the detection of fibrosis. The results in Figure 4 show that the Non-Alcoholic Fatty Liver Cirrhosis Disease Score (NCS) performed manifestly better than the other evaluated scores, with an AUROC of 0.85 (95% CI: 0.79, 0.91). TE was a close second and the most accurate imaging comparator for the detection of fibrosis (AUROC=0.8 (95% Ci: 0.76, 0.83)). LiMAx has a predictive accuracy of 0.77 and was the best at identifying fibrosis for patients of non-NAFLD aetiology. Remarkably, APRI performed equally as well as LiMAx with an AUROC of 0.76 (95% CI: 0.72, 0.8) and demonstrated a similar level of accuracy as seen for predicting a cirrhotic liver. Overall, the worst predictor is AAR (AUROC =0.66 (95% CI: 0.61, 0.71)), while the worst imaging comparator is spleen size (AUROC=0.69 (95% CI: 0.64, 0.74)), both of which are significantly poorer scores when assessed alongside LiMAx.

However, the final paper (Buechter et al. A) referred to it as “detecting CLD”. The decision to treat this as analogous to fibrosis was made as it is a semantic difference. This is due to the fact that, by definition, a diagnosis of CLD necessitates the presence of fibrotic changes. This paper also had some missing histology for their cohort but used other tests to confirm fibrosis.

Correlation Between LiMAx values and Biomarkers/Composite Scores

Jara et al. (2019) [22] and Malinowski et al. (2014) [27] reported the correlation values between LiMAx and other biomarkers (Table 3). After pooling the two studies, the degree of correlation between LiMAx and the four assessed parameters was seen to be extremely similar, with an absolute value ranging from 0.634 to 0.699. Bilirubin, MELD and CPS were negatively correlated; similarly, albumin also demonstrated a positive correlation. The confidence intervals are of approximately similar sizes, indicating comparable levels of variability within the sample. All pooled correlations have a p-value <0.05 and hence show statistical significance.

**Table 3:** Reported R values (Spearman’s rho) between LiMAx and biochemical parameters/ scores Pooled values, corresponding I^2^ and P values reported. MELD: Model for End-Stage Liver Disease; CPS: Child-Pugh Score; CI: confidence interval.

	Bilirubin	Albumin	MELD	CPS	
Pooled values	-0.699	0.634	-0.649	-0.674	
(95% CI)	(-0.837, -0.477)	(0.251, 0.846)	(-0.723, -0.561)	(-0.741, -0.595)	
p-Value	<0.001	0.003	<0.001	<0.001	
I^2^	95.10%	97.60%	70.00%	67.20%	

Correlation Between LiMAx and Histopathology of the Liver

Buechter et al. A (2019) and Buechter et al. B (2019) reported on correlation values between histological staging and LiMAx (Table 4) [24,25]. Pooling determined that LiMAx had the strongest (negative) correlation out of the contenders, with an r value of -0.75. All other tests had some degree of positive correlation as expected, but they were weaker than LiMAx. The closest was TE (0.69) and FIB-4 was the nearest non-imaging competitor at 0.6. All results were statistically significant.

**Table 4:** Reported R values (Spearman’s rho) between LiMAx and histological stages Pooled values, corresponding I2 and P values reported.

	LiMAx	TE	FIB-4	AAR	APRI
Pooled (95% CI)	-0.75 (-0.86, -0.60)	0.69 (0.54, 0.81)	0.6 (0.55, 0.65)	0.53 (0.44, 0.61)	0.47 (0.40, 0.53)
p-Value	<0.001	<0.001	<0.001	<0.001	<0.001
I^2^	86.20%	82.40%	0.00%	33.20%	0.00%

Discussion

The current review assessed the role of LiMAx in identifying fibrosis and cirrhosis compared to the existing composite scores and imaging tests. It suggests that LiMAX is the best predictor of cirrhosis and that LiMAx would be best utilised at predicting a cirrhotic liver with a pooled AUROC of 0.92, which was higher than all its non-invasive competitors.

CLD is one of the major rising causes of mortality in the UK [1]. Early diagnosis and appropriate management are crucial for CLD, as it can result in serious malignant and non-malignant complications. Measuring liver function accurately is the central pillar of management that underlies all therapeutic decisions. CLD is highly variable in its progression towards decompensation, with patients presenting at different times in the disease course. Currently, there are several well-validated options that act as a proxy for liver function, but they are accompanied by their own limitations, creating a clear gap for a direct measure of function such as LiMAx. Therefore, we aimed to compare the diagnostic and predictive performance of LiMAx with other tests. Participants of individual studies presented along the whole spectrum of the disease, but these dissimilarities atypically benefitted the review by allowing LiMAx’s predictive ability to be assessed as CLD progresses.

Out of the assessed outcomes, LiMAx would be best utilised at predicting a cirrhotic liver with a pooled AUROC of 0.92, which was higher than all its non-invasive competitors. Its ability to detect fibrosis was objectively lower (0.77), but it was still ranked the third highest in that meta-analysis and was the best for non-MASLD CLD patients. LiMAx is the highest scorer for cirrhosis, as it measures the maximum functional capacity of the liver, which dramatically reduces at the end stage of liver disease [29].

However, for 90-day mortality, LiMAx had an AUROC of 0.82, placing it firmly in the middle in comparison to the other tests. A variant of the test known as CreLiMax had the same predictive value as LiMAx, implying the addition of creatinine provided no extra value for predicting 90-day mortality. They combine patient characteristics for a detailed calculation. Nevertheless, LiMAx performed well against CPS and the other MELD-related scores.

To confirm LiMAx’s use for all CLD patients with uniform threshold levels, large-scale validation studies and investigations to satisfy the level of homogeneity of the disease process in different aetiologies need to be carried out with separate populations chosen by aetiology. Without this level of evidence, LiMAx cannot be responsibly integrated into clinical practice due to a potential for misdiagnosis. The biggest challenge this review faced was the lack of standardisation and presence of inter-study variability pervading several aspects of the analysis.

Firstly, the definitions of the milestones were ambiguous, particularly when considering the extent of fibrosis. Out of the three studies, only two clearly stated the use of histological evidence to establish the presence of fibrosis and act as a reference standard. The last study (Buechter et al. A) mentioned the use of histology where available; otherwise, a combination of ill-defined non-invasive measures acted as the reference standard [24]. In addition to this, the definitions of histological staging between each paper was ambiguous. Buechter et al. B [25] stated clearly within their methodology that biopsy samples were acquired from all patients and a blinded qualitative assessment was carried out according to the Desmet classification to grade the samples from F1 to F4. Buechter et al. A [24] outlined a less precise method, where they used histological evidence when available alongside other tests. However, there were similarities in both groups and both studies were carried out at the same time, with some of the same participants They cross-referenced each other in their studies and therefore, the decision to analyse the data jointly was made [24,25]. Secondly, a similar shortcoming is the paucity of a singular gold standard reference test throughout these studies identifying the true extent of disease, with studies admitting their classification was subject to change. However, this reinforces that CLD is intrinsically difficult to classify, with even the biopsy method having well-accepted limitations [25].

The differences in methodology were subtle and came from dissimilarity in outcome assessment. However, despite this suggesting that the studies may not be estimating the same quantity to affirm the presence of the outcome, it does not necessarily indicate that the true predictive ability differs [29,30]. In order to ascertain the actual impact of this heterogeneity, a separate review should be carried out. Another recommendation would be to include sufficient patients to facilitate subgroup analyses for the different aetiologies and stages of CLD to mitigate any potential biases from patient selection. LiMAx manufacturer’s state no such concerns, but due to its novelty, there is insufficient evidence to prove this and associated costs may be prohibitive.

We were unable to provide any threshold values for the sensitivities and specificities for each of the tests as several papers reported different values. Due to the seemingly arbitrary selection of cut-off values for each of the comparators, there was no statistical approach available that could reveal anything of significance, as the results would suffer from ‘threshold effects’ [19,30]. This means a change in threshold values would manipulate the sensitivity and specificity, rendering any comparisons invalid. This is not a failing specific to the study designs but due to the researcher’s subjectivity. This issue is a clear indicator of heterogeneous disease; however, this is unhelpful for the validation of new tests. Ideally, cut-off values require standardisation before these studies can be analysed together to provide definitive threshold values. As a direct result of this, the review was unable to discern any definitive threshold values for LiMAx for different clinical outcomes.

The current evidence concluded that LiMAx may be of use in the prediction of cirrhosis for CLD patients, as it outperforms all other conventional methods. LiMax also shows initial promise with early stages of disease and has use as an alternative diagnostic tool for morphological changes in CLD with a substantial AUROC value reinforced by its strong negative correlation, which is markedly stronger than other approaches. LiMAx is an obvious choice for predicting cirrhosis and would also be of benefit in less severe stages of disease when used in conjunction with other clinical tests.

## Conclusions

This review shows that LiMAx outperforms the existing non-invasive tests in predicting cirrhosis and is sensitive to changes in liver function that occur as a consequence of CLD. Its strength lies in a direct measurement of liver function and does not infer disease stage or mortality risk through surrogate markers that are susceptible to confounding factors.

However, widespread clinical adoption of LiMAx still requires large-scale, aetiology-specific validation and standardisation of threshold values. Current studies vary in reference standards and histological definitions, which introduces heterogeneity and limits direct comparison. For the integration of LiMAx into clinical practice, future research will need similar methodology, established thresholds and needs to be conducted in diverse patient groups reflective of the population with CLD. This could allow LiMAx to be a unique complimentary tool for staging CLD and guiding therapeutic decisions.
